# Innate T-αβ lymphocytes as new immunological components of anti-tumoral “off-target” effects of the tyrosine kinase inhibitor dasatinib

**DOI:** 10.1038/s41598-020-60195-z

**Published:** 2020-02-24

**Authors:** Alice Barbarin, Myriam Abdallah, Lucie Lefèvre, Nathalie Piccirilli, Emilie Cayssials, Lydia Roy, Jean-Marc Gombert, André Herbelin

**Affiliations:** 1 INSERM, 1082 Poitiers, France; 2 CHU de Poitiers, Poitiers, France; 3 Service d’Oncologie Hématologique de Thérapie Cellulaire, CHU de Poitiers, Poitiers, France; 4 INSERM CIC-1402, Poitiers, France; 5 Université de Poitiers, Poitiers, France; 6 Service d’Immunologie et Inflammation, CHU de Poitiers, Poitiers, France; 7 Service Clinique d’Hématologie, Hôpital Henri-Mondor, Créteil, France; 8 Université Paris-Est Créteil, Créteil, France

**Keywords:** Immunology, CD8-positive T cells, NKT cells

## Abstract

Kinase inhibitors hold great potential as targeted therapy against malignant cells. Among them, the tyrosine kinase inhibitor dasatinib is known for a number of clinically relevant off-target actions, attributed in part to effects on components of the immune system, especially conventional T-cells and natural killer (NK)-cells. Here, we have hypothesized that dasatinib also influences non-conventional T-αβ cell subsets known for their potential anti-tumoral properties, namely iNKT cells and the distinct new innate CD8 T-cell subset. In mice, where the two subsets were originally characterized, an activated state of iNKT cells associated with a shift toward an iNKT cell Th1-phenotype was observed after dasatinib treatment *in vivo*. Despite decreased frequency of the total memory CD8 T-cell compartment, the proportion of innate-memory CD8 T-cells and their IFNγ expression in response to an innate-like stimulation increased in response to dasatinib. Lastly, in patients administered with dasatinib for the treatment of BCR-ABL-positive leukemias, we provided the proof of concept that the kinase inhibitor also influences the two innate T-cell subsets in humans, as attested by their increased frequency in the peripheral blood. These data highlight the potential immunostimulatory capacity of dasatinib on innate T-αβ cells, thereby opening new opportunities for chemoimmunotherapy.

## Introduction

Protein tyrosine kinases (TK) are essential cellular signaling enzymes implicated in a variety of physiological processes such as proliferation, development, migration, apoptosis, metabolism, transcription and differentiation that are often dysregulated by tumorigenesis. Therefore, TK proteins constitute a privileged target for cancer therapy and numerous small molecule TK inhibitors (TKI) have been developed and used successfully to treat several types of cancers. Most approved drugs are type I and II inhibitors, which directly compete with ATP at the highly conserved ATP-binding site^1^, and are therefore prone to off-target effects. In oncology, TKI off-target effects might cause or contribute to the anti-tumoral activities of a compound. Nevertheless, direct TKI off-target effects might not fully explain the clinical success of these drugs in cancer treatment. Indeed, since the first TKI approval in 2001 (imatinib or Gleevec®, a TKI targeting the oncoprotein BCR-ABL in Philadelphia chromosome-positive leukemias), growing evidence indicates that the immune system has a major role both in determining its therapeutic efficacy and in restraining the emergence of escape mutations^2^.

Dasatinib, a second-generation TKI targeting the chimeric and oncogenic protein BCR-ABL, is also a potent Src kinase inhibitor with numerous other TK targets^3^, explaining its use in several clinical trials in combination with other drugs for the treatment of advanced solid cancers^4^. Most of the *in vitro* studies point toward an immunosuppressive effect^5–9^ of dasatinib, whereas *in vivo*, numerous immunostimulatory effects have been observed, both in mouse models and in patients treated with this TKI. For example, in melanoma, sarcoma, colon and breast cancer-bearing mice, dasatinib increases CD8 T-cells concomitantly with decrease in regulatory CD4 T-cells^10^. Recently, the same effect was observed in some patients with chronic myeloid leukemia (CML)^11^. In clinical use, immunostimulatory effects have been observed during long-term use of dasatinib^12–15^. The most striking of these effects is the induction of large granular lymphocytes (LGL), consisting of cytotoxic T lymphocytes and natural killer (NK) cells. This cell expansion is associated with better clinical outcomes in an appreciable proportion of BCR-ABL^+^ CML or acute lymphoblastic leukemia patients^15,16^. Another commonly known fact is the enhancement of NK cell functions under dasatinib treatment^17–21^.

However, aside from one study on T-γδ cells^13^, very little attention has been given to the effects of dasatinib on other important subsets of the immune surveillance of cancer: the unconventional T-cells exhibiting NK features^22^. This T-cell subset includes TCR-αβ cells such as the well-known invariant Natural Killer T-cells (iNKT). With their semi-invariant TCR, iNKT cells recognize antigens presented by the non-classical MHC-type I molecule CD1d and express high levels of the transcription factor PLZF (Promyelocytic leukemia zinc finger). Defects of iNKT cells in number and function, including a shift toward the Th2 phenotype, have been observed in several types of cancers in humans^23^. Accordingly, we have reported immune subversion of iNKT cells activities in CML patients at diagnosis^24,25^.

Recently, another unconventional TCR-αβ cell subset with NK-like properties has aroused our interest: innate CD8 T-cells, originally found in the mouse model (for review, see^26^). We identified this subset in healthy individuals as a new distinct CD8 T-cell subset characterized by the expression of killer-cell immunoglobulin-like receptors (panKIR/NKG2A) with a memory phenotype (high Eomesodermin (Eomes) expression) and prompt IFNγ production in response to the pro-inflammatory cytokines IL-12 and IL-18^27^. Like iNKT cells, innate CD8 T-cells are present in several tumors^26^, especially in CML with a reduced number and function at diagnosis but partial normalization in patients in remission under TKI therapy^28^. Furthermore, concomitantly with NK cell restoration, we showed supra-normalization of innate CD8 T-cell frequency in CML patients in treatment-free remission for over two years^29^.

iNKT cells and innate CD8 T-cells being candidates as key players in the immune-surveillance of cancer, we have hypothesized that they are potential targets of dasatinib. Using BALB/c mice because they contain more of these two unconventional subtypes of T-cells^26,30,31^, and peripheral blood material from BCR-ABL^+^ CML patients, we have demonstrated that dasatinib can influence both cell subtypes at functional and/or numerical level(s).

## Materials and Methods

### Study subjects and samples

Frozen peripheral blood mononuclear cells (PBMCs) from patients were obtained from the phase II DASA-PEGIFN clinical trial, registered with EudraCT number 2012-003389-42. Briefly, newly diagnosed BCR-ABL^+^ chronic phase CML patients started dasatinib 100 mg/day. Venous blood was collected at diagnosis and at 3 months after initiation of treatment. See Supplementary Table 1 for cohort description. All patients gave informed consent in accordance with the Declaration of Helsinki for participation in the study, which was approved by the scientific committee of the INSERM CIC-1402 (Poitiers, France) and Comité Protection Personnes Recherche Biomédicale Région Poitou Charentes (protocol number 12.10.31). PBMCs were isolated from blood samples by density gradient centrifugation (Histopaque®-1077, Sigma-Aldrich), resuspended in 90% fetal calf serum with 10% DMSO, and cryopreserved at −80 °C or in liquid azote until use.

### Experimental studies in animals

BALB/c Eomes-GFP transgenic mice were obtained after backcrossing C57BL/6 Eomes-GFP transgenic mice^32^ and wild-type BALB/c mice (Janvier Labs). All mice were bred and housed in specific-pathogen-free conditions in our animal facility (PREBIOS, Platform of Research and Experimentation in Health Biology of the University of Poitiers). All procedures were performed in accordance with the recommendations of the European Accreditation of Laboratory Animal Care and French institutional committee of Poitou-Charentes (COMETHEA, C2EA-84, n° 2016072216352833). Wild-type BALB/c mice were used for *in vivo* experiments and BALB/c Eomes-GFP transgenic mice were used for *in vitro* culture of splenocytes.

For oral gavage, dasatinib (Sprycel, BMS) was dissolved in water and administered at 20 mg/kg daily 5 days per week to 8-to-10-week-old female BALB/c wild type mice. After 8-weeks of oral gavage, spleen and thymus were harvested and cells either analyzed *ex vivo* by flow cytometry or cultured with IL-12 and IL-18 to assess IFNγ production as described below.

### Cell culture and functional assays

Splenocytes were isolated from eight-to-ten-week-old females and either analyzed *ex vivo* by flow cytometry or seeded in RPMI 1640 medium supplemented with 10% heat-inactivated FCS and antibiotics in 24-well plate at 2.10^6^ cells/mL. Splenocytes were cultured for 7 days in the presence of IL-15 (20 ng/ml; R&D Systems) with or without dasatinib (1 nM; Santa-Cruz Biotechnologies). For IFNγ production, IL-12 (20 ng/ml; R&D Systems) and IL-18 (20 ng/ml; MBL International) were added for the last 16 hours of cell culture, and Golgiplug (BD Biosciences) for the last 4 hours prior to analysis by flow cytometry.

### Flow cytometry

A detailed list of antibodies used to stain human and murine cells is provided in Supplementary Tables 2 and 3. For murine NKT identification, PE-conjugated murine CD1d tetramers loaded with PBS-57 were kindly provided by the National Institute of Health Tetramer Facility, Atlanta, GA. Briefly, dead cells were excluded using the Zombie (Aqua^TM^ or NIR^TM^) Fixable Viability kit (BioLegend), and then incubated 30 min with the appropriate antibody mix. For intranuclear and intracytoplasmic staining, cells were fixed and permeabilized with the anti-human Foxp3 staining kit according to the manufacturer’s protocol (eBioscience). Data were acquired on a FACs Verse cytometer with the FACSuite software (BD Biosciences) and analyzed using FlowJo v10 (TreeStar, Inc.). Gating strategies for human and murine immune cell subtypes are shown in Supplementary Figs. 6 and 7.

### Statistical analysis

Data are shown as means ± s.d, unless otherwise indicated in the figure legends. Differences between groups were determined either with paired two-tailed Wilcoxon test for human and *in vitro* mouse experiments or unpaired two-tailed Mann-Whitney test for *in vivo* mouse experiments, to calculate P-values, where *p < 0.05; **p < 0.01; ***p < 0.001 were considered statistically significant. NS, not significant. Sample number is indicated in each figure legend. Samples were not randomized, and investigators were not blinded to sample identities. All statistical data analyses were performed using GraphPad Prism 7 software (GraphPad software). Significant outliers were identified using the Grubbs’ test and excluded from analysis.

## Results

### Dasatinib drives activation of iNKT cells and promotes their Th1-like profile in mice

**Figure 1:**
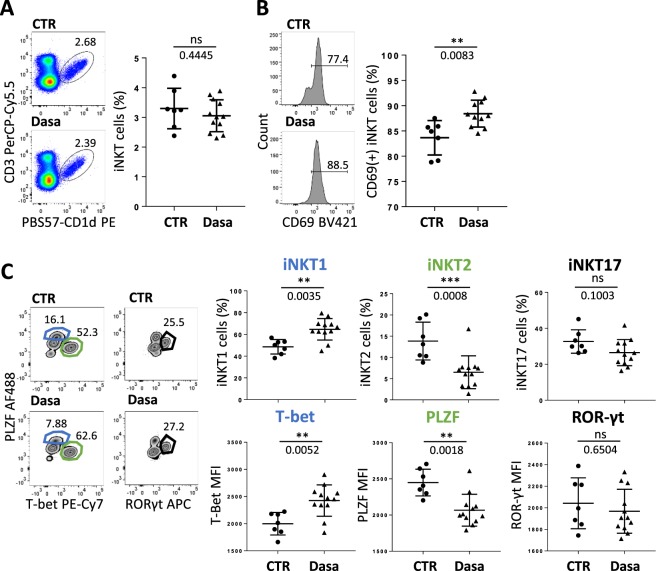
Dasatinib promotes type 1 iNKT cells in mice *in vivo*. (**A–C**) Flow cytometry analysis of thymic cells from BALB/c WT mice orally gaved with dasatinib (Dasa, n = 12) or its excipient (CTR, n = 8) for 8 weeks. Analysis of iNKT cell frequency (**A**) and CD69 positive iNKT cell frequency (**B**). (**C**) iNKT cell differentiation into NKT1, NKT2 and NKT17 subtypes: frequency (upper panel) and T-bet, PLZF and RORγt MFI in iNKT cells (lower panel) are shown. Representative plots are shown. Statistical analysis: Mann-Whitney two-tailed.

To determine the dasatinib effect on iNKT cells *in vivo*, BALB/c mice were orally given either dasatinib or its excipient 5 days a week for eight consecutive weeks. At the end of treatment, even though iNKT (CD3^+^PBS57-CD1d^+^) cell frequency in the thymus of BALB/c mice was not modified (Fig. 1A), the innate T-cell subset was found activated, as reflected by a higher CD69 expression level (Fig. 1B). We also analyzed dasatinib influence *in vivo* on iNKT cell differentiation into Th1, Th2 or Th17 subtypes, based on the expression level of the three transcription factors PLZF, T-bet and Eomes, respectively^33^. After dasatinib treatment, the thymus was enriched in iNKT1 (T-bet^+^ PLZF^int^) cells, depleted in iNKT2 (T-bet^−^ PLZF^high^) cells whereas the frequency of iNKT17 (PLZF^int^ RORγt^+^) cells remained unchanged (Fig. 1C).

To confirm this result, we analyzed the effect of dasatinib on iNKT cell homeostasis and functions in an *in vitro* splenocyte culture model. Precisely, isolated BALB/c splenocytes were cultured in the presence of IL-15 and with or without dasatinib. After 7 days, we found that dasatinib significantly increased the proportion of iNKT cells (Supplementary Fig. 1A). No change in the activation state and/or differentiation profile of iNKT cells was observed *in vitro* in response to dasatinib treatment, presumably because of the presence of IL-15 in all our culture conditions. Indeed, IL-15 is sufficient by itself to activate iNKT cells and drive them toward a Th1 (PLZF^int^ T-bet^+^) differentiation profile closely associated with IFNγ secretion (Supplementary Fig. 1B,C). Similar results were obtained with cultured splenocytes from the C57BL/6 mouse strain, ruling out a possible genetic background-dependent effect (**data not shown**).

### Dasatinib promotes iNKT cells in humans

**Figure 2:**
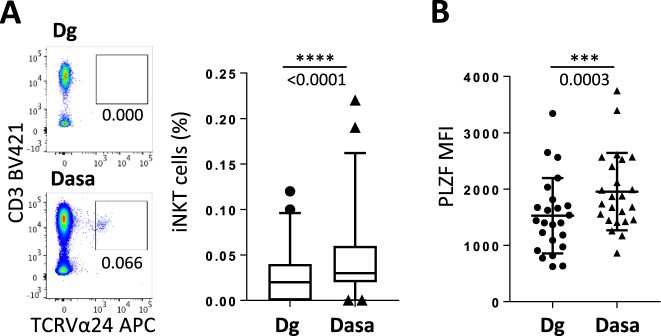
iNKT-cell frequency increases in CML patients under dasatinib treatment PBMCs isolated from patients (n = 47) at CML diagnosis (Dg) or after 3 months of dasatinib treatment (Dasa) were analyzed by flow cytometry for iNKT cells frequency (**A**) (box plot representation: median, quartiles, bars: 5 and 95 percentiles) and PLZF MFI in iNKT cells (n = 24) (**B**). Representative plots are shown. Statistical analysis: paired two-tailed Wilcoxon test.

We next extended our study to humans. Dasatinib is clinically used for the treatment of BCR-ABL^+^ leukemias, especially chronic myeloid leukemia (CML), because it blocks the deregulated tyrosine kinase ABL. Peripheral blood samples from newly diagnosed CML patients treated at first-line with dasatinib (see Methods and Supplementary Table 1) were analyzed at diagnosis and after 3 months of treatment. In this cohort of dasatinib-treated CML patients, iNKT cell frequency was increased after 3 months of treatment (Fig. 2A). This phenomenon was accompanied by a slight but significant increase in the proportion of the CD4^+^ CD8^−^ iNKT cell pool without affecting the double negative (CD4^−^ CD8^−^) iNKT cells (Supplementary Fig. 2A,B). However, we found an enhanced expression level of the specific transcription factor PLZF in the whole iNKT cell compartment (Fig. 2 and Supplementary Fig. 2), suggesting that dasatinib globally improves the functionality of iNKT cells without favoring a particular iNKT cell subtype.

### Dasatinib increases the frequency of mouse innate CD8 T-cells and promotes their IFNγ expression in response to an innate-like stimulation

**Figure 3:**
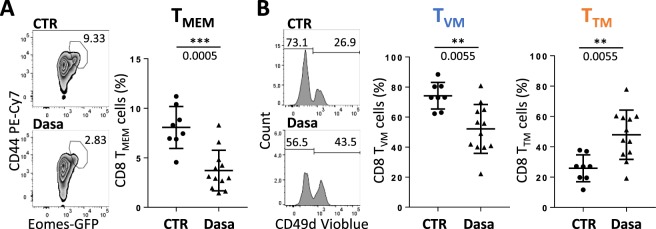
Dasatinib promotes CD8 T_TM_ cells in mice *in vivo*. Flow cytometry analysis of thymic cells from BALB/c WT mice orally gaved with dasatinib (Dasa, n = 12) or its excipient (CTR, n = 8) for 8 weeks. Analysis of CD8 T_MEM_ cells among CD8 T-cells (**A**) and population distribution between T_VM_ and T_TM_ cells among CD8 T_MEM_ cells (**B**). Representative plots and histograms are shown. Statistical analysis: two-tailed Mann-Whitney test.

Next, we analyzed the effect of dasatinib on innate-like CD8 T-cell development, using our *in vivo* model of oral dasatinib gavage. We found that total mature thymic CD8 T-cells (CD4^−^ CD24^−^ TCRβ^+^ CD8^+^) were not significantly affected by dasatinib (Supplementary Fig. 3). We focused our analysis on CD44^+^ Eomes^+^ CD8 T-cells, hereafter named T_MEM_ cells, that express a high level of CD122^26^ and can be considered as all innate-memory T-cells in the thymus. We found that their proportion was highly decreased by dasatinib treatment (Fig. 3A). However, when separately analyzing true memory CD8 T-cells (T_TM_, defined as CD44^+^ Eomes^+^ CD49d^+^ cells) and virtual-memory CD8 T-cells (T_VM_, defined as CD44^+^ Eomes^+^ CD49d^−^ cells)^26^, we found an inversion of the representation of the two innate-memory cell subtypes: after dasatinib treatment, the T_TM_ cell pool had highly increased whereas its T_VM_ counterpart had severely decreased (Fig. 3B).

**Figure 4:**
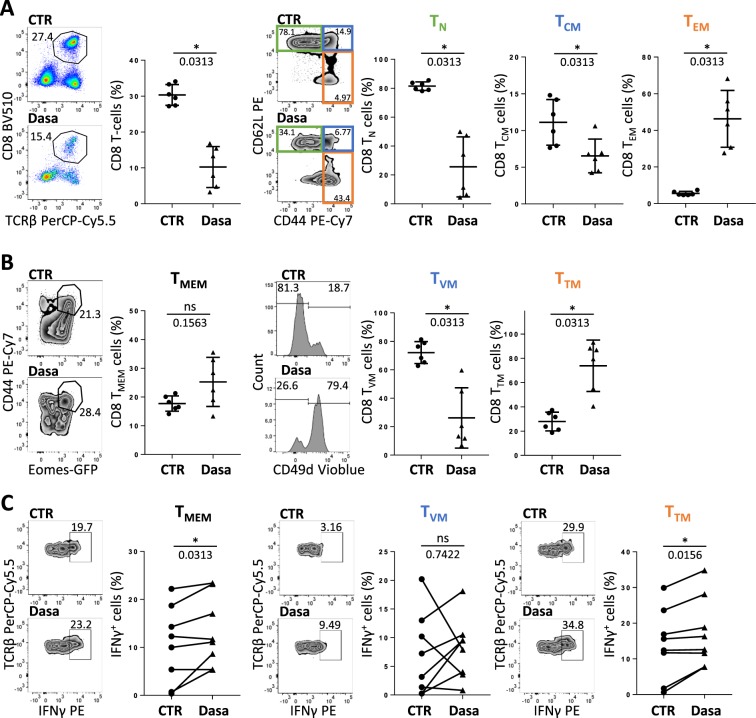
Dasatinib targets CD8 T_TM_ and CD8 T_VM_ cells in mice *in vitro*. (**A–C**) BALB/c Eomes-GFP derived splenocytes were cultured 7 days in the presence of IL-15 with (Dasa, n = 6) or without (CTR, n = 6) dasatinib, and analyzed by flow cytometry. (**A**) Analysis of total CD8 T-cells among live lymphocytes and population distribution between T_N_, T_CM_ and T_EM_ cells among CD8 T-cells. (**B**) Analysis of CD8 T_MEM_ cells among CD8 T-cells and population distribution between T_VM_ and T_TM_ cells among CD8 T_MEM_ cells. (**C**) Splenocytes were further stimulated for 16 h with IL-12 and IL-18 and IFNγ secretion was analyzed in T_MEM_, T_VM_ and T_TM_ cells (n = 8). Representative plots and histograms are shown. Statistical analysis: paired two-tailed Wilcoxon test.

To confirm the effect of dasatinib on innate-like CD8 T-cell homeostasis and functions, we used our *in vitro* cultured splenocyte model. Unlike *in vivo*, we observed a drastic diminution of the CD8 T-cell (TCRβ^+^ CD8^+^) compartment, along with modified cell subtype distribution. The proportions of both naive (CD44^−^ CD62L^+^) T-cells (T_N_) and central memory (CD44^+^ CD62L^+^) T-cells (T_CM_) decreased whereas the proportion of the effector memory (CD44^+^ CD62L^−^) T-cells (T_EM_) increased when dasatinib was added to cultures (Fig. 4A). As a result, the total memory CD8 T-cell compartment (T_MEM_), comprising T_CM,_ T_EM_ and innate-memory T-cells, was not significantly affected by dasatinib (Fig. 4B). As is the case with T_EM_ cells, the proportion of T_TM_ cells markedly increased in the presence of dasatinib, at the expense of T_VM_ cells (Fig. 4B). Similar data were obtained when applying the same experimental setting to C57BL/6 splenocytes, thereby ruling out a specific effect in the BALB/c genetic background (Supplementary Fig. 4A,B). To analyze the functional capacity of T_TM_ and T_VM_ cells, splenocytes were further stimulated with the pro-inflammatory cytokines IL-12 and IL-18. In agreement with our previous study^26^, we showed that among CD8 T-cells, secreted IFNγ in response to this innate-like stimulation arises mainly from innate CD8 T-cells with the T_TM_ profile^26^. Overall, we found that dasatinib induced a slight but significant increase of IFNγ secretion in the T_MEM_ compartment, especially in the T_TM_ compartment, as compared to its T_VM_ counterpart, the later showing unmodified levels of IFNγ secretion (Fig. 4C). Taken together, our *in vitro* and *in vivo* data led to the conclusion that in mice, dasatinib favors the development and functions of innate CD8 T-cells with a T_TM_ profile.

### Dasatinib promotes innate CD8 T-cells in humans

**Figure 5:**
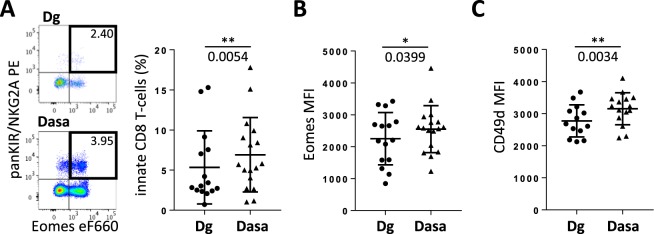
Innate CD8 T-cell frequency increases in CML patients under dasatinib treatment. PBMCs isolated from patients (n = 15) at CML diagnosis (Dg) or after 3 months of dasatinib (Dasa) treatment were analyzed by flow cytometry for panKIR/NKG2A^+^ Eomes^+^ innate CD8 T-cells (**A**). Eomes and CD49d MFI were analyzed in innate CD8 T-cells (**B**). Representative plots are shown. Statistical analysis: paired two-tailed Wilcoxon test.

Like iNKT cells, innate CD8 T-cells are severely reduced and functionally deficient in BCR-ABL^+^ CML patients at diagnosis^24,28^. By identifying innate CD8 T-cells in human peripheral blood as Eomes^+^ panKIR/NKG2A^+^ cells^27,28^, we found a significant increase of innate CD8 T-cell frequency in CML patients after 3 months of dasatinib treatment, as compared to values at diagnosis (Fig. 5A). Dasatinib enhanced the functionality of these cells by increasing Eomes expression level as well as the membrane protein CD49d, a surrogate marker of IFNγ secreting cells^26^ (Fig. 5B,C). However, the action of dasatinib on homeostasis of the CD8 T-cell compartment was not restricted to innate CD8 T-cells since the proportion of their conventional T_MEM_ (Eomes^+^ panKIR/NKG2A^−^) counterparts likewise increased while the frequency of naive-like (Eomes^−^ KIR^−^) CD8 T-cells concomitantly decreased (Supplementary Fig. 5).

## Discussion

TKI dasatinib is a widely used drug in cancer treatment and is well-known for its effects on the adaptive and innate immune systems, which probably contribute to its therapeutic value. However, the precise mechanisms of dasatinib action on the different parts of the immune system remain unclear. Deciphering dasatinib action on the immune system will help to better define the cancer situations in which the immune system could be aroused by dasatinib. Despite their potential anti-tumoral functions, little is known on dasatinib’s effects on unconventional T-αβ cell subsets, so we chose to study dasatinib’s effect on iNKT cells and innate CD8 T-cells. We treated tumor-free mice *in vivo* and splenocytes *in vitro* and showed that dasatinib promotes a Th1 profile in iNKT cells and increases CD8 T_TM_ cells in number and function. Then, starting with samples from a cohort of dasatinib-treated CML patients, we found that dasatinib also targets iNKT cells and innate CD8 T-cells in humans.

Previous studies have shown that dasatinib has effects on the immune system in preclinical mouse cancer models. A recent example is the study by Hekim *et al*., demonstrating that dasatinib exerted a pro-Th1 effect on iNKT cells with an increase of cytotoxic CD8 T-cells in peripheral blood and a decrease of CD4 regulatory T-cells in tumors^10^. While our study was conducted with tumor-free mice, we were similarly able to demonstrate a pro-Th1 differentiation of iNKT cells through increased T-bet expression. One possible mechanism is the action of dasatinib on PLZF expression level in thymic iNKT cells. Indeed, our data are consistent with the recent work of Park and al. showing that decreasing PLZF expression induced a higher number of NKT1 cell subtypes and a lower number of the NKT2 subtypes^34^. However, in this study, decreasing PLZF expression level was associated with an overall decrease of the pool of iNKT cells, an effect that we did not observe with dasatinib. One possible explanation is that dasatinib may act on other cell types to counterbalance this effect by inducing IL-15 secretion to sustain iNKT cell development. Indeed, it was shown by Powers *et al*. that surface IL-15 expression was increased on CD3^+^ CD57^+^ lymphocytes under dasatinib treatment^35^.

Contrasting with our results in mice, in CML patients treated with dasatinib, iNKT cells are higher in number, more activated and have an increased PLZF expression level. These results confirm a previous study by Rohon *et al*. showing an increase of the absolute NKT‐like cell counts in peripheral blood observed in dasatinib‐treated CML patients for a longer period (mean treatment time 11 months)^36^. However, for two reasons we could not confirm the existence of a pro-Th1 effect of dasatinib on iNKT cells in humans. First, iNKT cells are a rare population, especially in CML patients at diagnosis, and we could not assess IFNγ secretion function in these human samples. Second, in humans, iNKT cell subtypes are not as well-described as in the mouse, and it is generally admitted that DN (CD4^−^ CD8^−^) iNKT cells are in a more differentiated state than their CD4^+^ CD8^−^ counterparts. Recently, the CCR5 marker has been proposed to better discriminate undifferentiated iNKT cell subsets^37^, while Knox *et al*. suggested to use Eomes and T-bet transcription factor expressions concomitantly, showing low level T-bet expression in the more differentiated DN iNKT cells^38^. While our result showed that dasatinib did not affect the DN iNKT cell pool in terms of frequency, it could be interesting to investigate its possible effect on Eomes and T-bet expression levels in this iNKT cell subtype.

Dasatinib effects on CD8 T-cells in mouse models are not well-described. It has been shown that dasatinib inhibited antigen-specific proliferation of murine CD4 and CD8 transgenic T-cells *in vitro* and *in vivo*^9^. Several studies have been conducted in tumor models showing that the anti-tumoral effect of dasatinib involves CD8 T-cells, as increased levels of circulating tumor antigen-specific CD8 T-cells and a higher number of tumor-infiltrating CD8 T-cells have been observed^10,39,40^. To the best of our knowledge, our study is the first to investigate dasatinib’s effect on conventional and innate CD8 T-cells in a tumor-free mouse model. We found that dasatinib induced a drastic decrease of CD8 T_MEM_ cells with a shift toward T_TM_ cells rather than T_VM_ cells. *In vitro*, we showed that dasatinib favors T_TM_ cells and increases IFNγ secretion of CD8 T_MEM_ cells in response to IL-12+IL-18 pro-inflammatory cytokine stimulation. Further studies are needed to investigate the contribution of CD8 T_TM_ cells to the anti-tumoral effect of dasatinib.

An explanation for the observed decrease of CD8 T_MEM_ cells in the thymus is the effect of dasatinib on iNKT cells, and more specifically their decreased PLZF expression. Indeed, it was previously shown at steady state in several mouse models that PLZF-expressing T-cells, especially iNKT cells, promote the development of innate-memory CD8 T-cells, through their secretion of the Th2 cytokine IL-4^33,41–43^. The recent study by Park *et al*. supports this idea: decreased PLZF expression induced a lower number of innate CD8 T-cells (CD44^+^ CD122^+^) concomitantly with a decrease of both Eomes expression and IFNγ production in this cell compartment^34^. However, in this study the distribution between T_VM_ and T_TM_ (based on the expression of CD49d) was not observed. In our study, despite lowered PLZF expression in iNKT cells, dasatinib favored the T_TM_ cell subtype. Thus, we could not rule out the involvement of cytokines other than IL-4 to sustain T_TM_ cells. In this regard, it was recently shown in a systemic inflammatory mouse model induced by IL-12+IL-18 systemic production that innate CD8 T-cell development depends on both IL-4, IL-15 and IFNγ^44^.

Regarding our dasatinib-treated CML patient cohort, we observed an increase of the proportion of innate CD8 T-cells (Eomes^+^ panKIR/NKG2A^+^) among PBMCs. Aside from early work revealing the generation of LGL in dasatinib-treated patients^15,16^, very few studies have studied dasatinib action on CD8 T-cells. However, the study by Kreutzman *et al*., which showed that dasatinib treatment increased the numbers of granzyme B expressing memory CD4 and CD8 T-cells^12^, and the recent study by Wei *et al*. showing an increase of CD8 T-cells concomitantly with decreased Treg^11^, are in accordance with our own results showing an increased CD49d expression level on innate CD8 T-cells. Indeed, we have previously shown that innate CD8 T-cells have a higher cytotoxic potential than conventional CD8 T-cells^27,28^ and that the IFNγ secretion function is correlated with the expression of CD49d^26^. Our results are also in accordance with the study by Powers *et al*. showing a concomitant decrease of naive CD8 T-cells and increase of memory CD8 T-cells^35^. This led us to hypothesize that dasatinib may be able to promote the development of memory CD8 T-cells (conventional and innate) from the naive-like CD8 T-cell subset in humans. It is also to be noted that numerous studies have found that dasatinib enhances NK cell functions^17–21^. As innate CD8 T-cells share common features with NK cells, we hypothesize that dasatinib could act through the NKG2A or other KIR receptors to enhance the cytotoxicity of innate CD8 T-cells.

The precise mechanism by which dasatinib enhances the number and functions of iNKT cells and innate CD8 T-cells remains to be determined. As dasatinib directly interferes with Src kinases of the TCR signaling cascade^3^, it seems likely that like iNKT cells, due to a higher affinity for their ligand and a lower activation threshold^45^, unconventional T-cells are less impacted by dasatinib, and therefore have a more sustainable homeostasis/proliferation than conventional T-cells. Another possible mechanism is a cytokine microenvironment (IL-4, IL-15 and IFNγ) favoring unconventional T-αβ cell development induced by dasatinib, as discussed above in a mouse model. In humans, a plasma proteomic analysis of a small CML patient cohort treated 3 months with dasatinib revealed increased IFNγ and decreased IL-10 plasma levels. Moreover, IL-12 and IL-18 pro-inflammatory cytokines were both detected in plasma from dasatinib-treated patients^46^. As innate CD8 T-cells and iNKT cells are highly reactive to these two pro-inflammatory cytokines^27^, we can hypothesize an indirect mechanism through which dasatinib recruits iNKT cells and innate CD8 T-cells by stimulating IL-12 and IL-18 production. This idea is supported by the work by Goplen *et al*. demonstrating that IL-12 can transduce signals through the TCR pathway to support CD8 innate immune responses^47^, and also by the fact that dasatinib-mediated inhibition of T-cells does not induce apoptosis insofar as the effect is reversible or may be overcome by signals bypassing the TCR^7^.

To conclude, our study demonstrated new immune targets of dasatinib, namely iNKT cells and innate CD8 T-cells, cell subsets with potential anti-tumoral functions. As dasatinib immune off-target effects are used to enhance immune checkpoint therapy^48,49^ or to limit CAR-T cells therapy side effects^50^, its effects on unconventional T-cell immunity should be taken into account.

## Supplementary information

Supplementary Material.
