# Supplementary material for: Innate T-αβ lymphocytes as new immunological components of anti-tumoral “off-target” effects of the tyrosine kinase inhibitor dasatinib
Source: Sci Rep. 2020 Feb 24;10:3245. doi: 10.1038/s41598-020-60195-z (PMC7039999; doi:10.1038/s41598-020-60195-z)
Supplement: Supplementary file 1 — Supplementary Material. [file 41598_2020_60195_MOESM1_ESM.pdf]

SUPPLEMENTARY MATERIAL

Innate T-αβ lymphocytes as new immunological components of anti-tumoral "off-target" effects of the tyrosine kinase inhibitor dasatinib

Alice Barbarin[1,2], Myriam Abdallah[1], Lucie Lefèvre[1], Nathalie Piccirilli[1,2], Emilie Cayssials[1,2,3,4,5], Lydia Roy[7,8], Jean-Marc Gombert[1,2,5,6#] and André Herbelin[1,2,5,#,*]

[1]INSERM 1082, Poitiers, France

[2]CHU de Poitiers, Poitiers, France

[3]Service d'Oncologie Hématologique de Thérapie Cellulaire, CHU de Poitiers, Poitiers, France

[4]INSERM CIC-1402, Poitiers, France

[5]Université de Poitiers, Poitiers, France

[6]Service d'Immunologie et Inflammation, CHU de Poitiers, Poitiers, France

[7]Service Clinique d'Hématologie, Hôpital Henri-Mondor, Créteil, France

[8]Université Paris-Est Créteil, Créteil, France

#A.H. and J.M.G. contributed equally to this work as senior authors.

Corresponding author: André Herbelin[*]

Address: U1082 INSERM, Pôle Biologie Santé; 1, rue Georges Bonnet, 86000 Poitiers, France

Phone: (33) 5 49 45 43 41. Email: andre.herbelin@inserm.fr

Supplementary methods

Supplementary Table 1. CML Patients characteristics.

Group	n	Age range	Age median	Age mean	Sex ratio
DasaPEG	54	20-65	48	46	1.7

All patients were confirmed Philadelphia chromosome-positive.

Supplementary Table 2.
Anti-mouse antibodies used in this study.

Antibody	Fluorochrome	Clone	Supplier	Reference
CD69	BV421	H1.2F3	BD Biosciences	562920
CD3ε	PerCP-Cy5.5	17A2	BD Biosciences	100218
TCR-β	PerCP-Cy5.5	H57-597	BioLegend	109228
CD8	BV510	53-6.7	BD Biosciences	563068
CD44	PE-Cy7	IM7	BD Biosciences	560569
CD62L	PE	MEL-14	BD Biosciences	553151
CD49d	Vioblue	R1-2	Miltenyi	130-102-404
CD122	APC	TM-B1	BioLegend	123214
PLZF	AF488	Mags.21F7	eBioscience	53-9320-82
ROR-γT	APC	B2D	eBioscience	17-6981-82
T-bet	PE-Cy7	4B10	BioLegend	644823
panNK CD49b	APC-Cy7	DX5	BioLegend	108920
CD4	PE	RM4-5	BD Biosciences	553049
CD24	PE	M1/69	BD Biosciences	553262
Eomes	AF488	Dan11mag	eBioscience	53-4875
IFNγ	PE/PE-Cy7	XMG1.2	BD Biosciences	554412/557649

Supplementary Table 3.
Anti-human antibodies used in this study.

Antibody	Fluorochrome	Clone	Supplier	Reference
TCR-αβ	BV421	IP26	BioLegend	306722
CD3	BV421	UCHT1	BioLegend	300434
CD161	PerCP-Cy5.5	HP-3G10	BioLegend	339908
CD49d	BV510	9F10	BioLegend	304318
CD4	FITC	RPA-T4	BioLegend	300506
TCR Vα24-Jα18	APC	6B11	BioLegend	342908
CD8	PE-Cy7	RPA-T8	BD Pharmingen	557746
Eomes	eFluor®660	WD1928	eBiosciences	50-4877-42
PLZF	PE	P3.62.8.1	eBiosciences	12-4714-82
KIR2D	PE	NKVFS1	Miltenyi Biotec	130-092-688
KIR3DL1/KIR3DL2 (CD158e/k)	PE	5.133	Miltenyi Biotec	130-095-205
NKG2A (CD159a)	PE	REA110	Miltenyi Biotec	130-098-814

Supplementary Figure 1

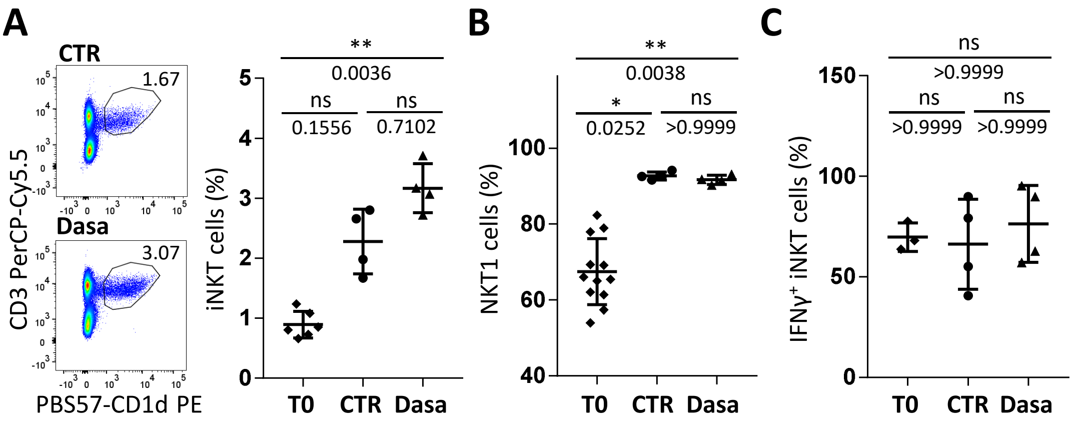

Supplementary Figure 1. IL-15 promotes Th1 differentiation of iNKT cells *in vitro*. (**A, B** and **C**) BALB/c Eomes-GFP derived splenocytes were directly analyzed (T0, n=12) or cultured 7 days in the presence of IL-15 with (Dasa, n=4) or without (CTR, n=4) dasatinib. iNKT cells percentages (**A**) and differentiation (**B**) into NKT1 subtype (T-bet^{+} PLZF^{int}) were analyzed in live lymphocytes by flow cytometry. (**C**) Splenocytes were further stimulated for 16h with IL-12 and IL-18 and IFNγ secretion was analyzed in iNKT cells. Statistical analysis: Kruskal-Wallis.

Supplementary Figure 2

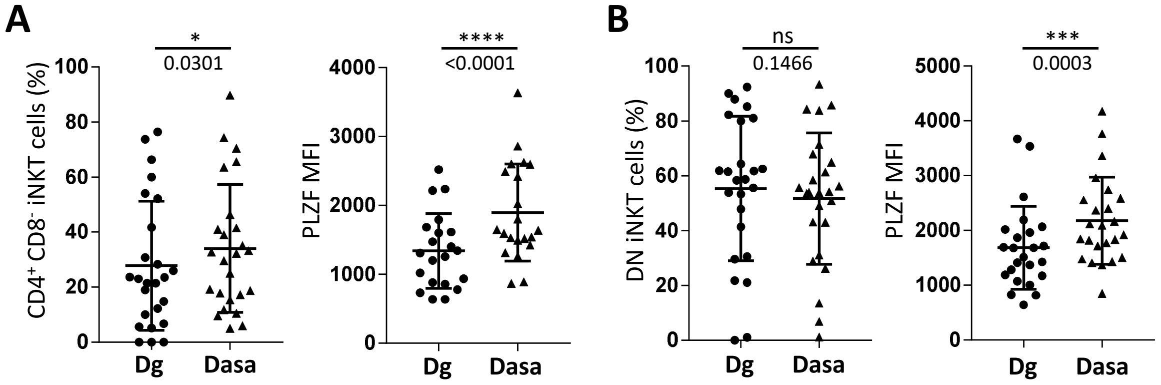

Supplementary Figure 2. In CML patients under dasatinib treatment, iNKT cells are increased. PBMCs isolated from patients (n=25) at CML diagnosis (Dg) or after 3 months of dasatinib treatment (Dasa) were analyzed by flow cytometry for iNKT cells. (**A**) Percentage of $CD4^{+}$ $CD8^{-}$ iNKT cells and PLZF MFI in this subset. (**B**) Percentage of DN ($CD4^{-}$ $CD8^{-}$) iNKT cells and PLZF MFI in this subset. Statistical analysis: paired two-tailed Wilcoxon test.

Supplementary Figure 3

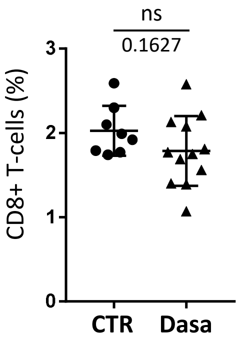

Supplementary Figure 3. Dasatinib does not affect the total CD8 T-cells compartment *in vivo*. Flow cytometry analysis of thymic cells from BALB/c WT mice orally gaved with dasatinib (Dasa, n=12) or its excipient (CTR, n=8) for 8 weeks. Analysis of TCRβ^{+} CD8^{+} T-cells among live mature lymphocytes (CD4^{-} CD24^{-}). Statistical analysis: two-tailed Mann-Whitney test.

Supplementary Figure 4

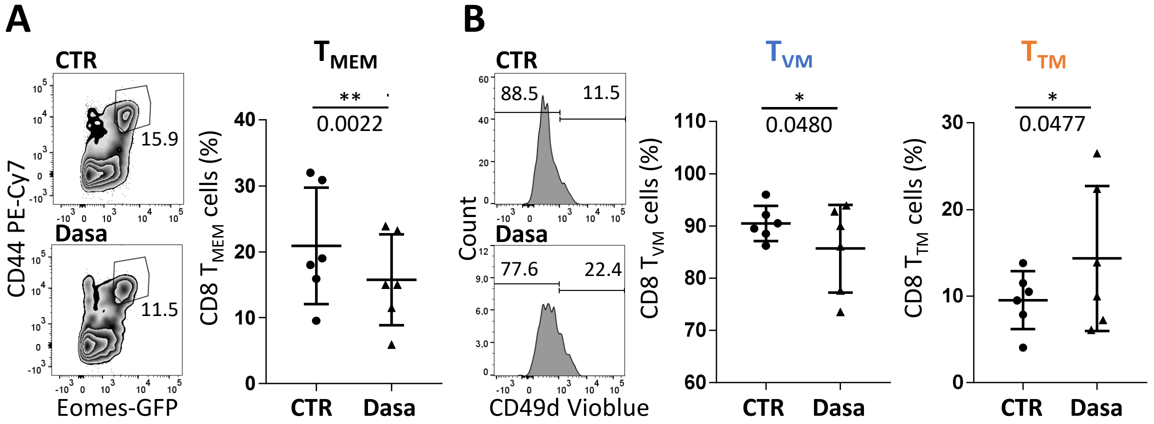

Supplementary Figure 4. Dasatinib targets CD8 T_{MEM} in C57BL6 splenocytes *in vitro*. (**A** and **B**) C57BL/6 Eomes-GFP derived splenocytes were cultured 7 days in the presence of IL-15 with (Dasa, n=6) or without (CTR, n=6) dasatinib, and analyzed by flow cytometry. (**A**) Analysis of CD8 T_{MEM} cells among CD8 T-cells. (**B**) Population repartition between T_{VM} and T_{TM} cells among CD8 T_{MEM} cells. Representative plots and histograms are shown. Statistical analysis: paired two-tailed or one-tailed t-test.

Supplementary Figure 5

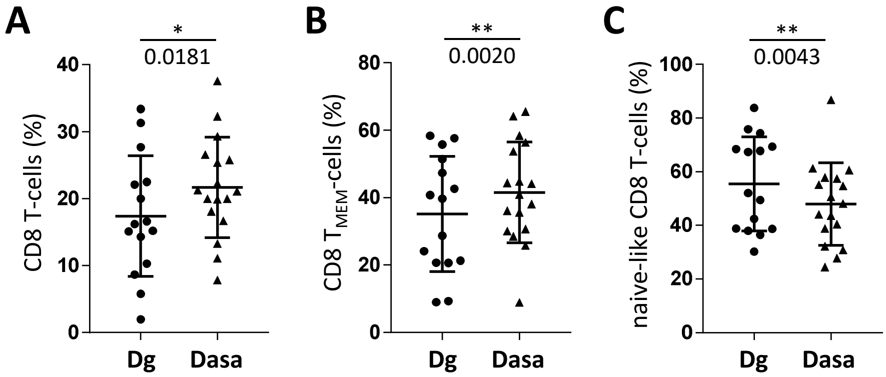

Supplementary Figure 5. CD8 T-cells subtypes repartition is modified in CML patients under dasatinib treatment. PBMCs isolated from patients (n=15) at CML diagnosis (Dg) or after 3 months of dasatinib treatment (Dasa) were analyzed by flow cytometry for CD8 T-cells (**A**) CD8 T_{MEM} cells (**B**) and naive-like CD8 T-cells. Statistical analysis: paired two-tailed Wilcoxon test.

Supplementary Figure 6

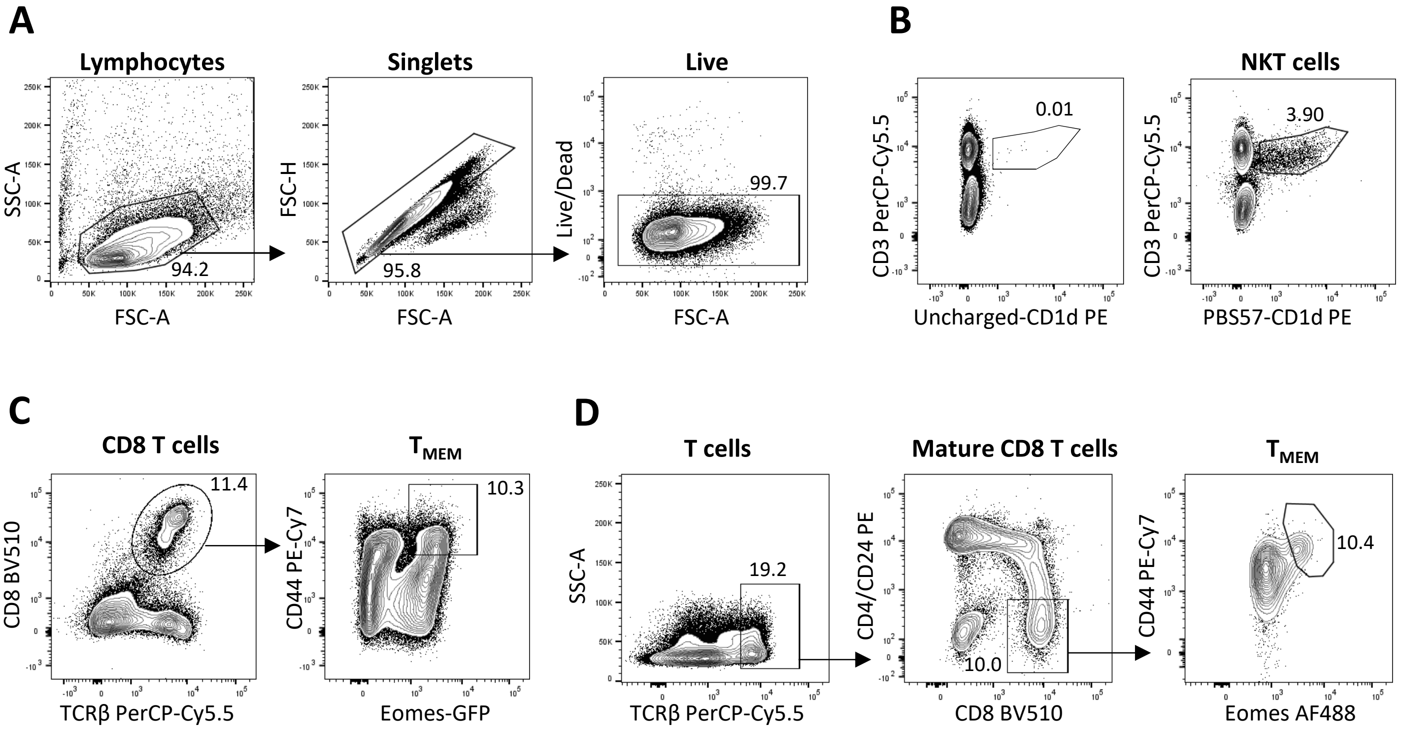

Supplementary Figure 6. Mouse gating trees for iNKT and T_{MEM} cells, related to Figures 1, 2 and 3. (**A**) Common gating tree to define live lymphocytes in spleen and thymus. (**B**) iNKT cells are defined as $CD3^{+}$ PBS57-$CD1d^{+}$ in spleen and thymus, uncharged CD1d tetramers were used as controls. (**C** and **D**) Gating tree for $TCR\beta^{+}$ $CD8^{+}$ T_{MEM} cells populations in spleen (**C**) and thymus (**D**) preparations from BALB/c WT or BALB/c Eomes-GFP mice, respectively, based on CD44 and Eomes expression. Representative plots from a 10 weeks-old mice are shown.

Supplementary Figure 7

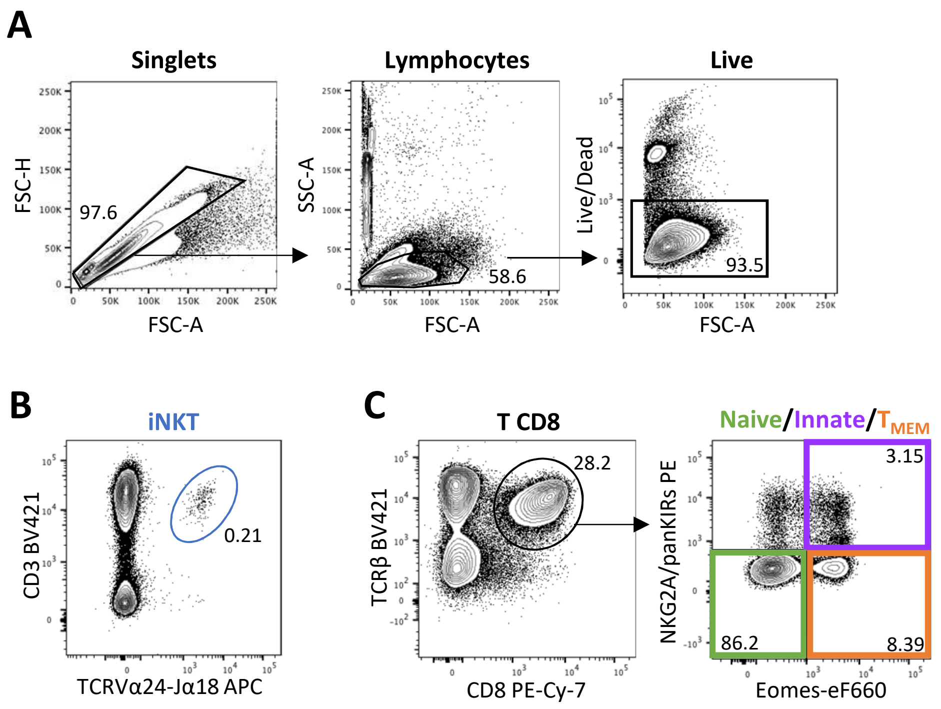

Supplementary Figure 7. Gating tree for iNKT, naive-like, T_{MEM} and innate CD8 T cells, related to Figure 4. Human gating tree to define live iNKT cells ($CD3^{+}$ $TCRV\alpha24\text{-}J\alpha18^{+}$) and live $TCR\beta^{+}$ $CD8^{+}$ naive-like, T_{MEM} and innate CD8 T cells populations in PBMC-derived preparations based on panKIR/NKG2A and Eomes expression. Representative plots from a 43 years-old human are shown.
